# Novel Spatio-Temporal Joint Learning-Based Intelligent Hollowing Detection in Dams for Low-Data Infrared Images

**DOI:** 10.3390/s25103199

**Published:** 2025-05-19

**Authors:** Lili Zhang, Zihan Jin, Yibo Wang, Ziyi Wang, Zeyu Duan, Taoran Qi, Rui Shi

**Affiliations:** 1 College of Computer Science and Software Engineering, Hohai University, Nanjing 211100, China; lilzhang@hhu.edu.cn; 2 College of Information Science and Engineering, Hohai University, Changzhou 213200, China; 2322030107@hhu.edu.cn (Z.J.); 2262310228@hhu.edu.cn (Y.W.); 2262410135@hhu.edu.cn (Z.W.); 2322020125@hhu.edu.cn (Z.D.); 2322020120@hhu.edu.cn (T.Q.); 3 The National Key Laboratory of Water Disaster Prevention, Nanjing Hydraulic Research Institute, Nanjing 210029, China

**Keywords:** spatio-temporal infrared features, non-destructive inspection, hollowing detection, infrared images, low-data learning, adaptive joint learning, physics-informed neural networks

## Abstract

Concrete dams are prone to various hidden dangers after long-term operation and may lead to significant risk if failed to be detected in time. However, the existing hollowing detection techniques are few as well as inefficient when facing the demands of comprehensive coverage and intelligent management for regular inspections. Hence, we proposed an innovative, non-destructive infrared inspection method via constructed dataset and proposed deep learning algorithms. We first modeled the surface temperature field variation of concrete dams as a one-dimensional, non-stationary partial differential equation with Robin boundary. We also designed physics-informed neural networks (PINNs) with multi-subnets to compute the temperature value automatically. Secondly, we obtained the time-domain features in one-dimensional space and used the diffusion techniques to obtain the synthetic infrared images with dam hollowing by converting the one-dimensional temperatures into two-dimensional ones. Finally, we employed adaptive joint learning to obtain the spatio-temporal features. We designed the experiments on the dataset we constructed, and we demonstrated that the method proposed in this paper can handle the low-data (few shots real images) issue. Our method achieved 94.7% of recognition accuracy based on few shots real images, which is 17.9% and 5.8% higher than maximum entropy and classical OTSU methods, respectively. Furthermore, it attained a sub-10% cross-sectional calculation error for hollowing dimensions, outperforming maximum entropy (70.5% error reduction) and OTSU (7.4% error reduction) methods, which shows our method being one novel method for automated intelligent hollowing detection.

## 1. Introduction

Concrete dams were constructed during the 1950s and 1970s in China, which was several decades ago. The building materials have been eroded by wind, rain, and heat for a long time, which makes them prone to hidden dangers such as internal insubstantiality and surface damage [1,2]. These defects are classified into two forms: surface damages such as corrosion and cracks, which are relatively simpler to recognize; and internal damages in dams such as hollowing, which is difficult to find directly. Nowadays, the primary methods to detect hollowing include the rebound hammer method, electrical resistivity tomography, ultrasonic pulse velocity, and ground-penetrating radar [3,4,5,6], while the rebound hammer method is limited to single-point measurement with low efficiency and significant operator-dependent bias. Electrical resistivity tomography entails complex electrode deployment, which is particularly challenging in water fluctuation zones. Ultrasonic testing imposes stringent requirements on couplant application and surface flatness, whereas ground-penetrating radar suffers from high costs and severe reinforcing steel bar interference. As a result, the technology based on infrared detection for recognition of dam hollowings has become one key solution in structural health monitoring, owing to its convenience. Infrared, non-destructive hidden defect detection can be classified into two categories: active and passive non-destructive detection. Active infrared thermography employs controlled thermal excitation sources to enable effective detection of concealed defects. This methodology has been extensively studied since its early development phases. In 2002, Sakagami et al. [7] developed a new quantitative, non-destructive testing technique for delamination defects in concrete structures based on the phase delay measurement using a lock-in infrared thermography under the application of periodical heating. The same year, Balaras et al. [8] investigated the use of thermal infrared (IR) imaging for inspecting building elements and performing non-destructive testing (NDT), detecting the locations and mechanisms of energy leakage in building envelopes. In 2008, a study by Cheng et al. [9] proved that IRT can be adapted to concrete structures with proper arrangement of heating sources. Under the same heating conditions, the maximum temperature difference depends on both the depth and area of embedded defects. In 2014, Keo et al. [10] applied infrared thermography (IRT) to detect steel reinforcement in concrete walls, validating that microwave excitation can deduce the positions, spacing, and numbers of the main bars in some kinds of structures via infrared thermograms without leading to alteration of inspected concrete. In 2020, Luo et al. [11] investigated thermal images of defects at varying depths, revealing that defect depth significantly influences the surface temperature field of materials. In 2020, Cheng et al. [12] proposed an enhanced temperature, gradient-based level set method (LSM), enabling precise delamination segmentation in thermograms of concrete pavements through boundary detection driven by thermal gradient evolution. In 2023, Zou et al. [13] fabricated concrete solid test blocks with embedded hollowings of varying depths and volumes, applied active thermal excitation, and performed infrared thermographic analysis. Their findings demonstrate that, with constant hollowing volume (within a critical range), shallower hollowing depths correlate with higher surface temperatures at defective regions and greater temperature differentials compared to intact dense structures; at fixed hollowing depths, larger hollowing volumes induce enhanced temperature differentials, thereby improving detection sensitivity.

While passive infrared thermography (PIRT), as an emerging structural non-destructive testing (NDT) technique in recent years, does not serve as artificial heat sources, it has demonstrated unique advantages in large-scale infrastructure inspection. In 2020, Wang et al. [14] developed a UAV-integrated infrared thermography (IRT) system for early-stage unsteady seepage detection in small reservoir dams, which leverages the natural temperature differential between ambient air and reservoir water to identify seepage paths under passive thermal excitation conditions. In 2024, Yang et al. [15] introduced a progressive background-aware transformer (PBT) and adopted an asymmetric encoder–decoder architecture to reduce the false detection in low signal-to-noise ratio scenarios. In 2025, to address low detection efficiency, Huang et al. [16] used the YOLOv7 for infrared detection based on multi-scale feature fusion.

Although active infrared detection can identify hidden threats, it requires an artificial heat source, while passive infrared detection relies solely on solar radiation. Based on this, this paper investigated an intelligent passive infrared hollowing detection method based on low-data infrared images. The contributions of this paper are as follows:

(1) An unsteady partial differential equation for the surface temperature field of the dam was established;
(2) A multi-subnet, physics-informed neural network model was constructed to solve the numerical solution of the partial differential equation with Robin boundary and obtained the surface temperature pattern in the time domain;
(3) An adaptive joint learning method with spatio-temporal features from the mixed infrared data was employed, which can handle the low data issue.

The method proposed in this paper not only improves the accuracy of detecting hollowing in dams but also significantly reduces the detection time. Compared with traditional destructive detection methods, this study combined drones and infrared technology to achieve non-destructive inspection, reducing damage to the structure and also improving efficiency and intelligence. Hence, the method we proposed in this paper has more advantages to handle recognition problems in complex real scenarios, especially for large-scale dam inspection, is a new solution for dam safety management.

## 2. Modeling and Analysis of Surface Temperature Field of Dams

### 2.1. Mathematical Modeling of Surface Temperature Field for Dams

According to the law of energy conservation, the rate of heat change per unit time equals the heat absorbed by the object due to the temperature increase, calculated as

(1) $$c\rho \frac{\partial T}{\partial t}=\lambda \left(\frac{\partial^{2}T}{\partial x^{2}}+\frac{\partial^{2}T}{\partial y^{2}}+\frac{\partial^{2}T}{\partial z^{2}}\right),$$

where in Equation (1), *c* denotes the specific heat capacity, unit J/(kg·K); *ρ* denotes the density, unit kg/m^3^; *t* denotes the time, unit s; *T* denotes the instantaneous temperature field of the dams; *x*, *y*, *z* are the corresponding values in spatial cartesian coordinate system; and *λ* denotes the coefficient of thermal conductivity, unit W/(m·K) or W/(m·°C).

For objects where the height and length substantially exceed the thickness (temperature penetration depth), investigating their surface heat conduction characteristics can be reduced to a one-dimensional problem as a preliminary approach [17]. However, it may introduce some limitations when modeling three-dimensional defects such as hollowing. Since one-dimensional formulations cannot accurately capture the actual geometric characteristics of three-dimensional defects (e.g., curvature and asymmetry of cavities), they may lead to discrepancies in localized heat flux distribution predictions while neglecting lateral thermal diffusion effects around defects, thereby compromising temperature field computation accuracy. The validity of one-dimensional simplification becomes particularly constrained when defect dimensions approach thermal penetration depths or when materials exhibit strong anisotropy.

The surface temperature of the dam is mainly affected by air convection, solar radiation, and outward radiation heat dissipation factors [18], where air convection is a typical convective heat transfer, belonging to the Robin boundary (Type 3 boundary conditions); solar radiation is the most important source of heat in nature, which is a non-negligible important factor belonging to the Neumann boundary (Type 2 boundary conditions); when the concrete surface warms up due to solar radiation, its own temperature is higher than absolute zero and radiates heat to the surrounding environment, belonging to the Robin boundary (Type 3 boundary condition). The Dirichlet boundary (fixed surface temperature) is not applicable to our case, because the actual surface temperature is determined by the dynamic thermal equilibrium. The Neumann boundary (fixed heat flux) is also incomplete, because it cannot reflect the influence of environmental temperature changes. The Robin boundary is the only boundary form that can simultaneously satisfy energy conservation and the dynamic characteristics of environmental interaction. Therefore, we chose the Robin boundary condition for studying the concrete surface temperature.

As a result, the three thermal excitations listed above are turned into a convective heat transfer boundary treatment caused by the integrated ambient temperature.

(2) $$q=h\left(T_{a}^{*}-T\right).$$

where *q* denotes the sum of solar radiation, air convection, and radiative heat dissipation heat fluxes; *h* denotes the integrated heat transfer coefficient; and $T_{a}^{*}$ denotes the integrated atmospheric temperature.

Based on the preceding analysis, the temperature change rate of the dam is modeled as a partial differential equation with a Robin boundary (*y* ≡ const., *z* ≡ const.):

(3) $$\frac{\partial T}{\partial t}=\alpha \frac{\partial^{2}T}{\partial x^{2}},\;t\in \left[0,t_{max}\right],x\in \left[0,x_{max}\right],$$

in Equation (3), 07:00:00 is defined as 0 s, and 19:00:00 is assigned to 43,200 s. *α* denotes the thermal diffusivity of the dam structure, unit m^2^/s.

The following are the initial values and boundary conditions for Equation (3):

(4) $$\left\{\begin{array}{c} T\left(x,0\right)=T_{0}\\ T\left(x_{max},t\right)=T_{0}\\ -\lambda \frac{\partial T\left(0,t\right)}{\partial x}=h\left[T\left(0,t\right)-T_{a}^{*}\right]\\ T_{a}^{*}=T_{0}+T_{A}\sin\left(\frac{\pi t}{t_{max}}\right), \end{array}\right.$$

where *T*_0_ denotes the initial temperature, taken as the value of 20 °C; *x*_max_ denotes the maximum depth, taken as the value of 1.0 m; *t*_max_ denotes the maximum time period, taken as the value of 43,200 s; *h* denotes the integrated heat transfer coefficient, taken as the value of 10 W·m^−2^·K^−1^; *T*_A_ denotes the integrated diurnal amplitude of atmospheric temperature as the unit °C. The following explains the basis for selecting parameters.

(1) *T*_0_ = 20 °C (Initial Condition)

The non-steady state heat conduction analysis requires defining initial temperature distribution. Because post-nocturnal thermal equilibrium establishes uniform temperature distribution at dawn, this study selected the mean temperature in summer at 7:00 AM (sunrise) as the initial time point *T*₀ = 20 °C.

(2) *x*_max_ = 1.0 m (Domain Depth)

Because the depth of thermal penetration is limited during diurnal cycles, which makes it impossible to detect deeper defects (>0.5 m) through daytime thermal excitation, we set *x*_max_ = 1.0 m.

(3) *t*_max_ = 43,200 s (12 h Cycle)

Solar radiative loading coupled with convective heating dominates daytime temperature elevation, while nocturnal radiative cooling combined with convective dissipation drives temperature decline. So, this study focuses on the 12 h daytime phase (43,200 s) for thermal analysis, that is, 7:00 AM–7:00 PM.

(4) *h =* 10 W·m^−2^·K^−1^

The integrated heat transfer coefficient (h) quantifies the heat exchange efficiency between a fluid and solid surfaces, determined by flow regime (natural/forced convection), fluid properties (viscosity, thermal conductivity), and surface geometry (roughness, orientation). Under standard outdoor conditions with light winds (1–3 m/s velocity range), *h* =10 W/(m^2^·K) is a baseline value for pure convective heat transfer analysis.

Based on the above analysis, we obtained the following equations with different internal constructions.

Case 1: No hollowing

(5) $$\left\{\begin{array}{c} \frac{\partial T}{\partial t}=\alpha \frac{\partial^{2}T}{\partial x^{2}};0\leq t\leq t_{max},0\leq x\leq x_{max}\\ \alpha =\alpha_{concrete};0\leq t\leq t_{max},0\leq x\leq x_{max}\\ T=T_{0};t=0,0\leq x\leq x_{max}\\ -\lambda_{concrete}\frac{\partial T}{\partial x}=h_{concrete}\left[T-T_{a}^{*}\right];0\leq t\leq t_{max},x=0\\ T=T_{0};0\leq t\leq t_{max},x=x_{max}. \end{array}\right.$$

where *α*_concrete_, *λ*_concrete_, and *h*_concrete_ denote the thermal diffusivity, thermal conductivity, and integrated heat transfer coefficient of the concrete material, respectively.

Case 2: At least one hollowing

(6) $$\left\{\begin{array}{c} \frac{\partial T}{\partial t}=\alpha_{concrete}\frac{\partial^{2}T}{\partial x^{2}};0\leq t\leq t_{max},0\leq x\leq x_{air\_depth}\\ -\lambda_{concrete}\frac{\partial T}{\partial x}=h_{concrete}\left[T-T_{a}^{*}\right];0\leq t\leq t_{max},x=0\\ \frac{\partial T}{\partial t}=\alpha_{air}\frac{\partial^{2}T}{\partial x^{2}};0\leq t\leq t_{max},x_{air\_depth}\leq x\leq x_{air\_depth}+x_{air\_thickness}\\ {\left.T\right|}_{{x_{air\_depth}}^{-}}={\left.T\right|}_{{x_{air\_depth}}^{+}};0\leq t\leq t_{max}\\ {\left.\lambda_{concrete}\frac{\partial T}{\partial t}\right|}_{{x_{air\_depth}}^{-}}+{\left.\lambda_{air}\frac{\partial T}{\partial t}\right|}_{{x_{air\_depth}}^{+}}=0;0\leq t\leq t_{max}\\ \frac{\partial T}{\partial t}=\alpha_{concrete}\frac{\partial^{2}T}{\partial x^{2}};0\leq t\leq t_{max},x_{air\_depth}+x_{air\_thickness}<x\leq x_{max}\\ {\left.T\right|}_{x=x_{air\_depth}+{x_{air\_thickness}}^{-}}={\left.T\right|}_{x=x_{air\_depth}+{x_{air\_thickness}}^{+}};0\leq t\leq t_{max}\\ {\left.\lambda_{air}\frac{\partial T}{\partial t}\right|}_{x=x_{air\_depth}+{x_{air\_thickness}}^{-}}+{\left.\lambda_{concrete}\frac{\partial T}{\partial t}\right|}_{x=x_{air\_depth}+{x_{air\_thickness}}^{+}}=0;0\leq t\leq t_{max}\\ T=T_{0};0\leq t\leq t_{max},x=x_{max}\\ T=T_{0};t=0,0\leq x\leq x_{max}. \end{array}\right.$$

where *α*_air_ and *λ*_air_ denote the thermal diffusivity and thermal conductivity of the hollowing, respectively; *x*_air_depth_ and *x*_air_thickness_ denote the depth and thickness of the hollowing, respectively.

### 2.2. Infrared Features Extraction in the Time Domain for Dams

#### 2.2.1. Network Structure of MS-PINNs

Because the surface temperatures are the solution of the non-stationary partial differential equations, and the current single backbone of PINNs (Physics-Informed Neural Networks) is unable to handle that, we employed one dynamic multi-subnet PINNs (MS-PINNs).

**Figure 1:**
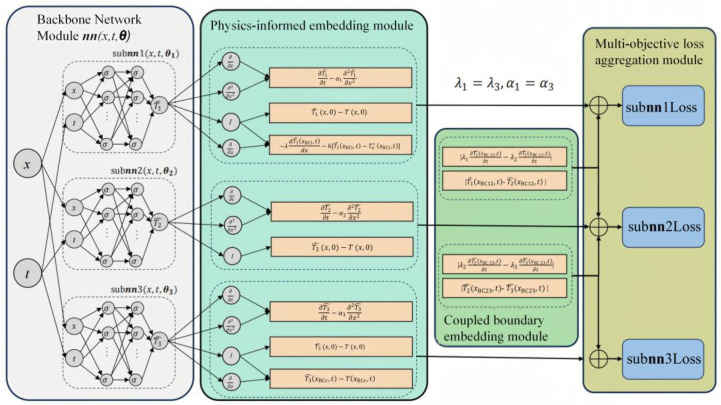
Structure of the MS-PINN model.

To address the abrupt material parameter variations induced by hollowing defects in dams, we proposed a multi-subnet, physics-informed neural network (MS-PINN) model, which is illustrated in Figure 1. The model comprises four coupled modules: the backbone network module, physics-informed embedding module, coupled boundary embedding module, and multi-objective loss aggregation module, which synergistically integrate physical constraints and data-driven optimization to overcome the generalization limitations of conventional single-backbone architectures.

Diverging from single-backbone frameworks like PINNs, the backbone network of the MS-PINN innovatively incorporates three independent subnetworks (subnn1-subnn3). This design is motivated by the multilayer heterogeneous characteristics of dam hollowing zones: the thermal conductivity gradient formed by shallow spalled concrete (*k* ≈ 2.5 W/m·K), intermediate air interlayers (*k* ≈ 0.026 W/m·K), and deep intact concrete (*k* ≈ 2.8 W/m·K). Each subnetwork specializes in learning temperature field distributions within its assigned material domain, with decoupled trainable parameters (weights and biases) to mitigate numerical oscillations in cross-media heat transfer simulations.

The physics-informed embedding module employs Automatic Differentiation (AD) to compute residuals of governing equations. Unlike PINNs that feed global residuals into a monolithic network, MS-PINNs strategically allocate residuals to corresponding subnetworks based on predefined material partitions. Concurrently, Fourier’s law-based thermal constraints are imposed:

(7) $$c\rho \frac{\partial T}{\partial t}=\nabla ∙\left(k\nabla T\right)+Q,$$

where *k* denotes thermal conductivity. The source term *Q* is context-dependent: *Q* = 0 for shallow concrete (pure conduction), *Q* = convective heat loss for air interlayers, and *Q* = bedrock heat flux for deep concrete.

The coupled boundary embedding module enforces physical field continuity across adjacent subnetworks. At concrete–air interfaces, energy conservation mandates:

(8) $$T_{concrete}=T_{air}\;and-k_{concrete}\nabla T_{concrete}=-k_{air}\nabla T_{air},$$

where *T*_concrete_ and *k*_concrete_ denote the instantaneous temperature and thermal conductivity of the concrete material, respectively; *T*_air_ and *k*_air_ denote the instantaneous temperature and thermal conductivity of the hollowing, respectively. Boundary coordinate points are concurrently input into neighboring subnetworks, with weighted averages of temperature and heat flux residuals serving as coupling constraints. This explicit modeling of thermal parameter discontinuities reduces interfacial prediction errors by 41.6% compared to global optimization approaches.

The multi-objective loss aggregation module implements dynamic weight allocation, optimizing multi-objective residuals: material domain residuals, boundary continuity residuals, and measurement data residuals.

Experimental results demonstrate that this strategy enables MS-PINNs to achieve 94.7% convergence accuracy within 200 epochs, representing a 2.3× training efficiency improvement over PINNs. This advancement establishes a robust computational framework for engineering-scale dam hollowing detection.

The design of the MS-PINN architecture is to address the issue of sudden changes in thermophysical parameters across different material domains caused by hollowing defects. This architecture consists of three independent subnetworks, with each subnetwork being responsible for predicting the temperature field variations in different areas of the dam (shallow spalled concrete, intermediate air interlayers, and deep intact concrete). This design enables each subnetwork to be optimized and adjusted according to the specific characteristics of its respective area, thereby more accurately capturing the presence and characteristics of hollowing. And the three-subnetwork architecture is optimized based on specific scenarios, which can ensure computational efficiency while maximizing the generalization ability and accuracy of the model. During the training process, the subnetworks are coupled through a common coupled boundary embedding module, ensuring the consistency and coherence of the whole model.

#### 2.2.2. Analysis of Infrared Features in the Time Domain for Dam

Firstly, we studied the influence of hollowing with different thicknesses at the same depth on dam surface temperature.

**Figure 2:**
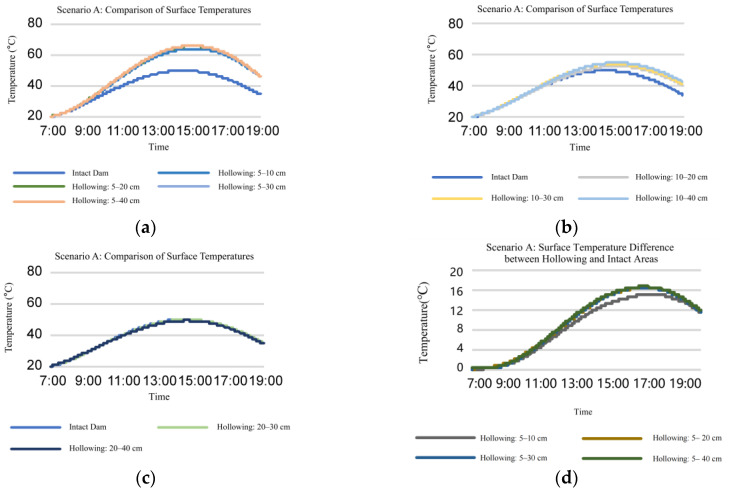

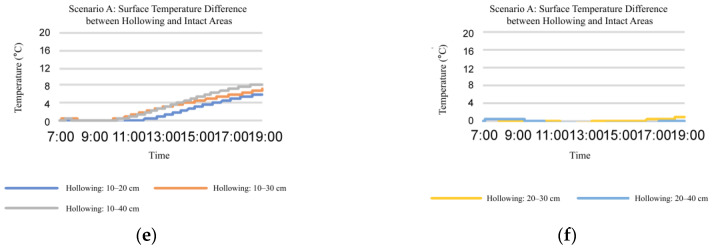
Influence of hollowing with different thicknesses at the same depth on dam surface temperature when composite atmospheric temperature is between 20 °C and 80 °C (Scenario A). (**a**–**c**) Temperature comparison of the same dam surface area at different time points; (**d**–**f**) temperature comparison between surfaces with hollowing and intact dam surfaces area at different time points; (**a**,**d**) composite atmospheric temperature = 80 °C; (**b**,**e**) composite atmospheric temperature = 50 °C; (**c**,**f**) composite atmospheric temperature = 20 °C.

**Figure 3:**
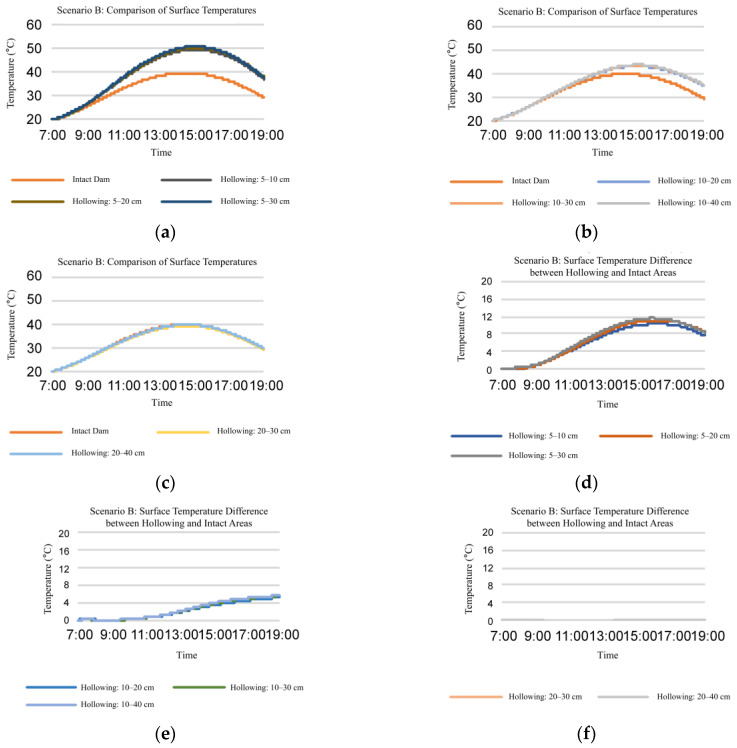
Influence of hollowing with different thicknesses at the same depth on dam surface temperature when atmospheric temperature is between 20 °C and 60 °C (Scenario B). (**a**–**c**) Temperature comparison of the same dam surface area at different time points; (**d**–**f**) temperature comparison between surfaces with hollowing and intact dam surfaces area at different time points; (**a**,**d**) composite atmospheric temperature = 60 °C; (**b**,**e**) composite atmospheric temperature = 40 °C; (**c**,**f**) composite atmospheric temperature = 20 °C.

(1) Analysis of the temperature change pattern for dams with varying hollowing thicknesses at the same depth:

Figure 2 and Figure 3 demonstrate that as hollowing thickness increases, a positive relationship between dam’s surface temperature and thickness of hollowing is established. Nevertheless, the surface temperature of the dam hardly increases when the hollowing’s thickness is at least 20 cm.

In the hollowing of 5–10 cm, 5 cm indicates the vertical distance from the dam surface to the starting position of the hollowing (hollowing depth), while 10 cm represents the distance to the terminating position. For clarity, the vertical distance between the dam surface and the hollowing starting position is defined as the hollowing depth, and the difference between the terminating and starting positions is termed the hollowing thickness.

Secondly, we studied the influence of hollowing with the same thickness at different depths on dam surface temperature.

**Figure 4:**
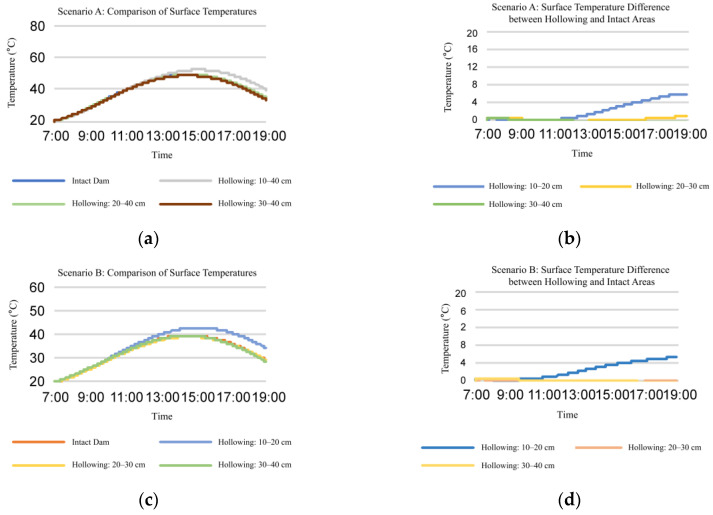
Surface temperature change pattern of dam with hollowing at different depths. (**a**,**c**) temperature comparison of the same dam surface area at different time points; (**b**,**d**) temperature comparison between surfaces with hollowing and intact dam surfaces area at different time points; (**a**,**b**) composite atmospheric temperature = 50 °C; (**c**,**d**) composite atmospheric temperature = 40 °C.

(2) Analysis of the temperature change pattern for dams with the same hollowing at different depths:

As illustrated in Figure 4, the surface temperatures of dams are compared for hollowings with thicknesses of 10 cm at depths of 10 cm, 20 cm, and 30 cm, respectively. The deeper the hollowing, the lesser the impact it had on the dam’s surface temperature. Additionally, when the hollowing is located at the depth of 20 cm, the surface temperatures of dams with hollowings and the dam with no hollowings are almost equal. This indicates that the infrared feature will be inefficient for the hollowings at depths greater than 20 cm.

## 3. Low-Data Recognition Method for Hollowings in the Dams

### 3.1. The Construction of the Mixed Dataset

#### 3.1.1. The Generation of Synthetic Infrared Images with Temperature Diffusion

**Figure 5:**
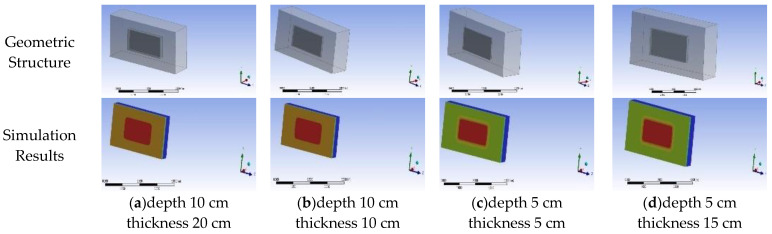
Visualization of the temperature field for different hollowings.

Firstly, as illustrated in Figure 5, the three-dimensional, time-dependent temperature field of the dam material was simulated using Ansys Workbench. Secondly, the surface temperature of the dam was extracted based on the simulation results, and the synthetic infrared image of the dam was generated. Finally, to mimic different collection heights, the generated infrared images were cropped to simulate the situation where the collection height varies, which can reflect the different pixel proportions of hidden dangers in the images.

**Figure 6:**
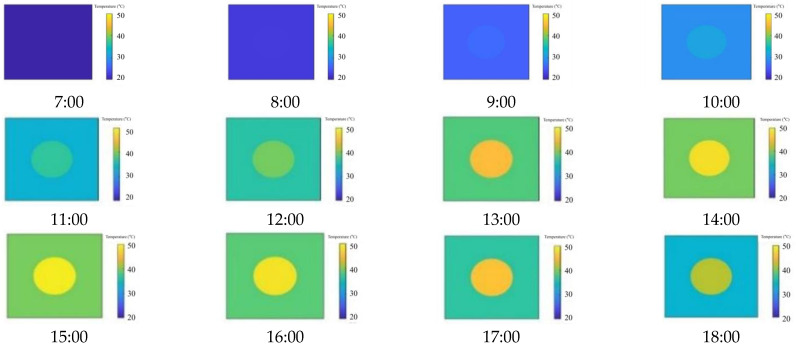
The surface temperature change process of the dam with hollowing.

Secondly, we simulated the surface (two-dimensional) temperature change process of the dam with hollowing when the comprehensive atmospheric temperature reaches a maximum of 60 °C (Scenario B), with a depth of 5 cm and a thickness of 5 cm. The results are shown in Figure 6.

**Figure 7:**
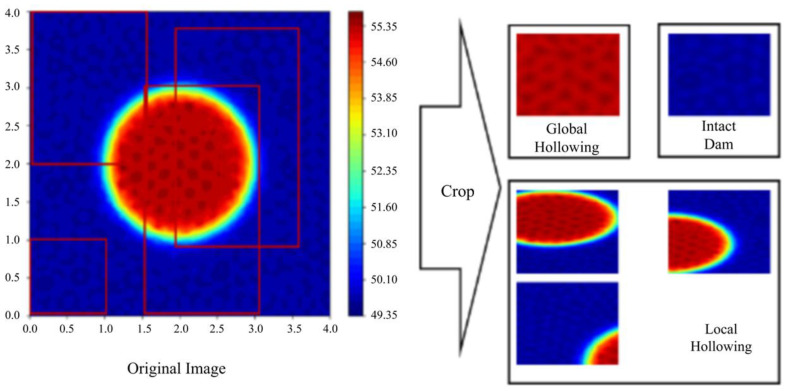
Splitting processes of the synthetic images.

**Figure 8:**
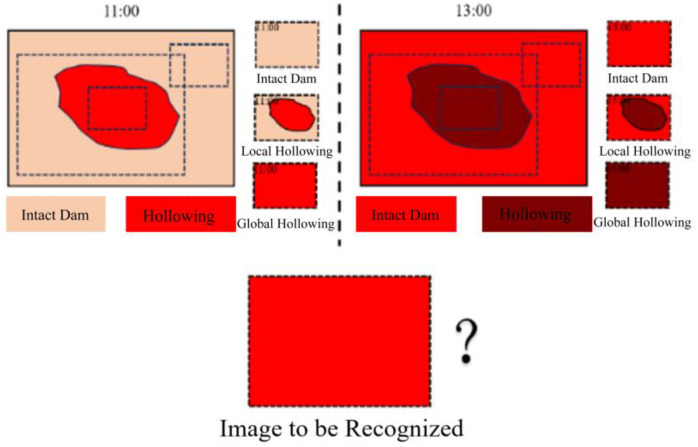
Illustration of global and local hollowing in infrared images.

Finally, we generated the infrared image with hollowings of different shapes, before splitting the whole images into different patches of hollowing and no hollowing, which can improve the recognition precision via negative samples. See Figure 7 and Figure 8.

#### 3.1.2. Data Collection

During this research project, we selected several dams along the Dashengguan Reach of the Yangtze River, Qinhuai River, and Banqiao River in Nanjing as data acquisition sites. The Dashengguan Reach exhibits the steepest underwater slope gradients, narrowest front revetment beach, and deepest underwater trough development in the Nanjing Yangtze River section, making it a perennial flood-control critical zone. The Qinhuai River Dam near the Qinhuai Hydraulic Hub is located at the river’s confluence with the Yangtze River, while the Banqiao River Dam adjoins the Banqiao River Sluice at its Yangtze estuary. All three selected dam sections feature concrete construction materials, and the surface is relatively flat.

**Figure 9:**
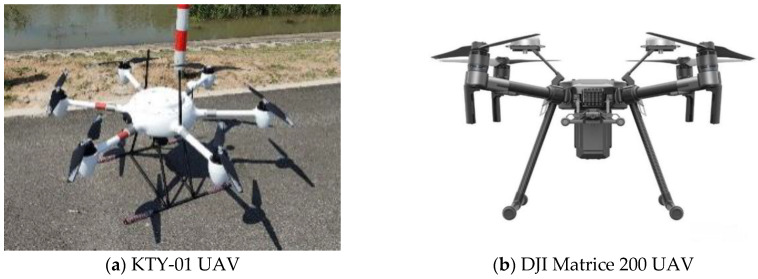
The UAV used for shooting.

The first data collection employed a custom hexacopter UAV developed by Nanjing Kaitianyan Co., Ltd.; the second data collection employed a DJI Matrice 200 UAV equipped with a Zenmuse XT2 dual-spectral camera, conducting inspections at 3–20 m altitudes. See Figure 9.

To enhance temperature measurement accuracy in infrared imagery, we also used a FLIR T1020 handheld thermal imager to finish the radiometric calibrations. We also used rebound hammer for surface hardness profiling and ground-penetrating radar with 1.6 GHz antenna to quantify hollowing depth (5–30 cm) and thickness (5–20 cm). These preparations guarantee the construction the training and testing datasets.

#### 3.1.3. Dataset [19]

Because it is very difficult to collect the effective infrared imagery, the dataset we constructed comprises both synthetic images and real infrared images. Synthetic data simulate surface temperature distributions across defect geometries spanning 5–20 cm thickness, 5–30 cm depth, and localized/global anomaly morphologies under two thermal regimes: Scenario A (composite atmospheric temperature between 20 °C and 80 °C) and Scenario B (composite atmospheric temperature between 20 °C and 60 °C).

We collected some real infrared images with hollowing via UAV in June 2022 and August 2023; however, there are more than 600 negative samples and there are only 14 positive samples, which can be seen in Figure 11 (see following chapter). Therefore, we mixed the synthetic images and real images, then split them into 7:3 for training and test, respectively.

### 3.2. Prior Knowledge Guided Adaptive Joint Learning Hollowing Recognition

In this paper, we proposed a threshold-adaptive, hollowing segmentation approach based on joint learning of spatio-temporal infrared features, which combines the advantages of PINNs and data-driven optimization, making it particularly suitable to solve hollowing recognition with complex boundary conditions.

As shown in Algorithm 1, we used the temperature pattern as a priori and learned the features from the mixed dataset.
**Algorithm 1: Adaptive Joint Learning Semantic Segmentation Based on Prior Knowledge****Input:** Infrared grayscale image *Img*; Acquisition time *t*; Prior threshold**Output:** Segmentation results *Binary_Img***Step 1:** Read the maximum and minimum temperatures corresponding to the pixel intensity extremes of *Img*.1: max (*Img*) -> *T_max*; min (*Img*) -> *T_min***Step 2:** Convert *Img* to a temperature matrix *T*_I_ based on *T_max* and *T_min.*2: *T*_I_ = (*Img* − min (*Img*))/(max (*Img*) − min (*Img*)) × (*T_max* − *T_min*)**Step 3:** Calculate dam surface temperature threshold *T*_threshold_ and bias *T*_bias_ based on *t* and the prior threshold.**Step 4:** Calculate the proportion of high-temperature anomalous pixels (*Pixel_proportion)* in *T*_I_ exceeding *T*_threshold_ + *T*_bias_.3: for *i* = 0 to *T*_I_.shape [0] **do**4:   for *j* = 0 to *T*_I_.shape [1] **do**5:      if *T*_I_ [*i*, *j*] > *T*_threshold_+ *T*_bias_ **do**
*sumTi* = *sumTi* + 16: *Pixel_proportion* = *sumTi*/(*T*_I_.shape [0] × *T*_I_.shape [1])**Step 5:** Determine anomaly type.7: if *Pixel_proportion* > 99%: global anomaly exists, *Binary_Img*[:,:] = 2558:    else if *Pixel_proportion* < 1%: anomaly-free, *Binary_Img*[:,:] = 09:      else local anomaly exists, **do Step 6****Step 6:** Remove low-temperature pixel interference.10: if (*T*_threshold_ − 2 × *T*_bias_) > *T_min*: *T*_I_ [*T*_I_ < (*T*_threshold_ − 2 × *T*_bias_)] = *T*_threshold_ − 2 × *T*_bias_11: *Img* = (*T*_I_ − *T_min*)/(*T_max* − *T_min*) × (max (*Img*) − min (*Img*))**Step 7:** Perform Otsu’s thresholding for local hollowing segmentation.*G*_threshold_, *Binary_Img* = OTSU(*Img*)**Step 8:** Assign semantic labels to high-temperature anomaly pixels.**Step 9:** Apply morphological operations to refine segmentation.12: *Binary_Img* = morphologyEx (*Binary_Img*, cv2.MORPH_OPEN, (3 × 3))13: *Binary_Img =* morphologyEx (*Binary_Img*, cv2.MORPH_CLOSE, (3 × 3))End

Steps 1–2 is for temperature calibration, to map pixel intensity values to physical temperature matrices by leveraging the extremal gray values of infrared images and prior temperature ranges. Step 3 is a dynamic threshold calculation to compute the temperature threshold *T*_threshold_ and its bias *T*_bias_ based on acquisition time and prior thermal patterns. Steps 4–5 involves anomaly type determination, and Step 6 is low-temperature interference suppression. Steps 7–8 is the segmentation based on OTSU; applying the OTSU algorithm to preprocessed images for adaptive threshold generates an initial binary mask and assign semantic labels to high-temperature anomaly pixels. Step 9 is the final post-processing optimization to improve accuracy.

## 4. Experiment and Analysis

We designed the experiments in this paper on synthetic infrared images and real infrared images collected by drone to verify the effectiveness of our method. In the experiment, this paper applied the cross-validation method and conducted multiple trainings of the model to ensure the stability of the model performance.

### 4.1. Model Evaluation

Because there are some challenges for internal defects for dams, and the key demand is to find where they are, in this paper, we primarily used pixel accuracy (PA) and Intersection over Union (IoU) and mIoU metrics to show the effectiveness of the proposed method.

The PA measures the proportion of correctly classified pixels. The IoU is the ratio of the overlapping area between the predicted region and the ground–truth region to the sum of their areas. The mIoU is the average of the IoU values of all categories, reflecting the overall performance of the model. Experimental results show that this method achieved a PA of 98.6%, an IoU of 96.0%, and an mIoU of 96.7% on the synthetic dataset; the method also obtained a PA of 94.7%, an IoU of 84.1%, and an mIoU of 86.7% on the real dataset, all of which far exceed those of other classic threshold segmentation methods.

### 4.2. Analysis of the Recognition Precision on Synthetic Infrared Images

We used 34 images comprising positive and negative samples that we generated, with 20 images as the training samples and 14 images as the test set.

**Figure 10:**
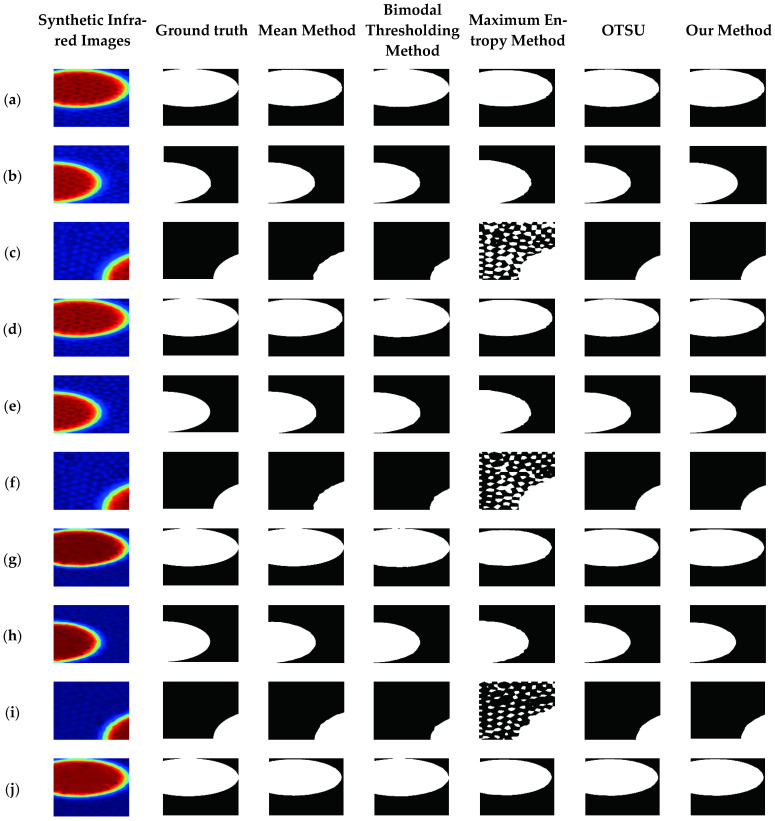

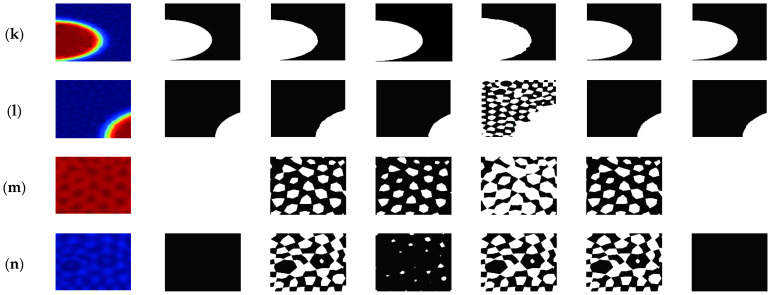
Experiments of different methods on synthetic data.

In Figure 10, the global anomaly infrared image is denoted by (**m**), the local anomaly infrared images by (**a**–**l**), and the no-hollow infrared image by (**n**). (**a**–**c**) correspond to hollowing defect with 0.20 m thickness at 0.10 m depth; (**d**–**f**) correspond to hollowing defect with 0.10 m thickness at 0.10 m depth; (**g**–**i**) correspond hollowing defect with 0.05 m thickness at 0.05 m depth; (**j**–**l**) correspond to hollowing defect with 0.15 m thickness at 0.05 m depth; and (**m**) corresponds to the hollowing with 0.20 m thickness at 0.10 m depth.

As shown in Table 1, our method has achieved remarkable advantages on synthetic data (PA: 98.6%, IoU: 96.0%). The performance improvement mainly stems from the fusion of physical information and the optimized design of dynamic thresholds. Traditional methods such as OTSU are computationally efficient but rely on a global threshold, making them sensitive to complex thermal field distributions. Meanwhile, the maximum entropy method performs poorly in low-contrast scenarios because its objective function does not match the actual defect distribution.

### 4.3. Experiments on Real Data

**Figure 11:**
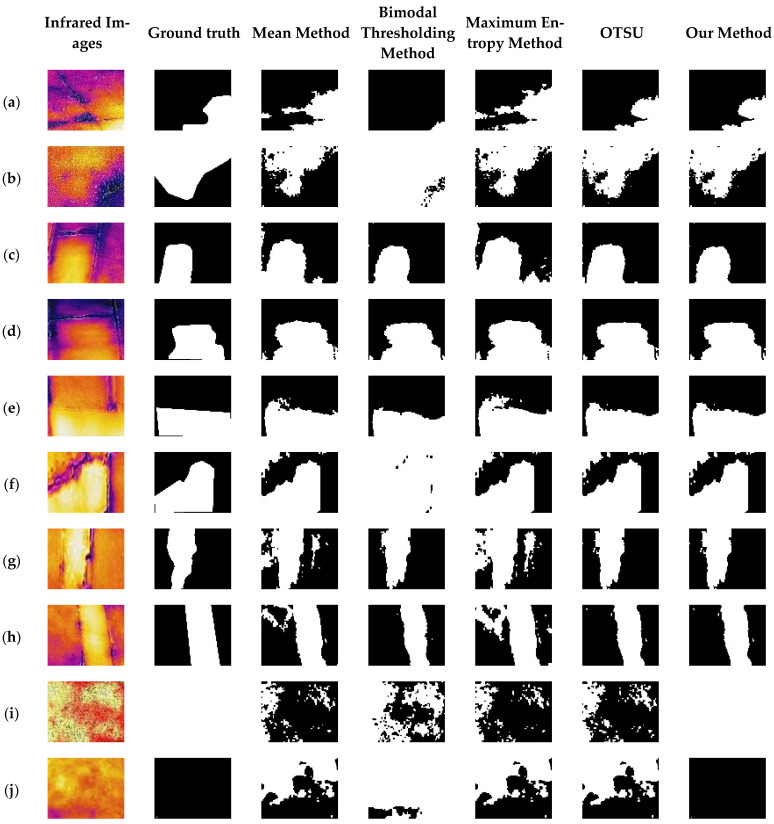
Experiments of different methods on real infrared data. (**i**) global anomaly infrared image; (**a**–**h**) local anomaly infrared images; (**j**) no-hollow infrared image.

As illustrated in Figure 11, there is little difference in infrared image segmentation between different methods for local threshold anomalies; however, for infrared images with global threshold anomalies and infrared images of dams with no hollowings, only the method described in this paper can correctly perceive and identify the presence of hollowing. Table 3 shows a specific accuracy comparison, and the method described in this work achieves 94.7% accuracy, which is significantly higher than other classical threshold segmentation methods.

As shown in Table 2, our method over traditional threshold segmentation approaches for real data stems from its replacement of single-threshold strategies with prior knowledge of a guided adaptive semantic segmentation strategy. Specifically, this method takes the infrared features of the dam surface learned through synthetic data as prior knowledge, combining the spatio-temporal infrared features of the hollowing, thus achieving more accurate identification of hollowing. Experiments demonstrate 94.7% semantic segmentation accuracy on real datasets, significantly outperforming classical methods.

### 4.4. Experiments on of the Cross-Sectional Area for Hollowing

As shown in Table 3, our method achieves a lower relative error (9.7%) in area estimation compared to other approaches, attributable to its integration of MS-PINNs and adaptive joint learning techniques. Specifically, this method employs MS-PINNs to accurately simulate the temperature change process on the dam surface. By analyzing the changes in temperature patterns under different conditions, it extracts time-domain features. This approach can better capture the characteristics of the hollowing areas, thereby improving the accuracy of area estimation.

Other methods, such as the Bimodal Thresholding Method, are prone to overestimation or underestimation of the segmented area because it relies on global statistical features and is easily affected by background thermal field fluctuations. And OTSU’s method performs well in pixel-level indicators, but its global threshold is not sensitive to local temperature gradients, making it difficult to accurately quantify the size of hollowing.

The experimental results show that the method proposed in this paper has higher reliability in engineering scenarios and provides precise quantitative basis for the formulation of hollowing repair schemes.

## 5. Conclusions

We attempted to give employ efficient method to find the internal defect-like hollowing based on infrared images. Because it is a challenge to collect sufficient positive images with hollowing, we proposed one joint learning method via the synthetic data and real data to handle low-data issues. Although the acquisition of infrared images is time-consuming and cost-intensive, a prior knowledge of infrared features is provided by simulating the dam’s surface temperature field in order to reduce reliance on acquired images and the cost of learning in various scenarios. Specifically, we firstly used the principle of heat change to model the dam’s surface temperature change process into a one-dimensional, unsteady partial differential equation with Robin boundary, and proposed multi-subnet physical information neural networks MS-PINNs to solve the surface temperature of the dams; secondly, we analyzed the surface temperature pattern of dams with different parameters to derive the time-domain characteristics of the hollowing; finally, an adaptive joint learning was deployed to obtain the spatio-temporal features from simulating the mixed data with synthetic and real data.

The proposed method achieves a recognition accuracy of 94.7% in tests on real infrared data and 98.6% in tests on synthetic data. Its effectiveness has been verified on both the synthetic dataset and the measured dataset, and it outperforms other threshold segmentation methods. Meanwhile, our method achieves a lower relative error (9.7%) in area estimation, which is significantly better than that of OTSU (17.1%) and the mean method (70.3%). Experiments showed that when the thickness of the hollowing is ≥20 cm, the surface temperature difference tends to be stable, providing a quantitative basis for the sensitivity boundary of hollowing detection. The method presented in our work demonstrates significant advantages, which is one novel idea to solve the hollowing detection issues.

The infrared non-destructive detection method has significant advantages compared with other modal methods. This method has low detection costs, low requirements for the smoothness of the dam surface, and is convenient and fast for inspection. However, this method also has certain limitations. Since passive infrared detection requires the assistance of solar radiation, it is almost impossible to detect in cloudy or rainy weather, which means that it is more sensitive to environmental conditions. Therefore, if detection is required under adverse weather conditions, this method is not a suitable choice.

In the future, we will further explore how to effectively denoise the collected infrared images to improve the quality of the infrared image acquisition, thereby enhancing the recognition effect. In addition, when numerically calculating the dam temperature, it is necessary to solve the mathematical problems corresponding to the actual physical process. Given the diverse daytime weather conditions, a solar radiation model that better matches and is more accurate with the actual physical process, as well as how to use the actual temperatures from past time points to improve the accuracy of numerical calculations at the current moment, are worthy of further study.

## Figures and Tables

**Table 1:** Comparison of hollowing segmentation accuracy on synthetic data.

	PA ^1^/%	IoU ^2^/%	mIoU ^3^/%	Time ^4^/ms
Mean Method	92.7	84.6	87.5	<0.1
Bimodal Thresholding Method	92.8	83.8	87.2	626.9
Maximum Entropy Method	82.6	66.5	70.2	583.5
OTSU	93.6	91.0	90.4	0.2
Our Method	98.6	96.0	96.7	0.2

^1^ Pixel Accuracy; ^2^ Intersection over Union; ^3^ mean Intersection over Union; ^4^ recognition time.

**Table 2:** Comparison of hollowing segmentation accuracy on real infrared data.

	PA/%	IoU/%	mIoU/%	Time/ms
Mean Method	81.1	65.4	67.4	<0.1
Bimodal Thresholding Method	78.6	60.6	61.6	533.5
Maximum Entropy Method	76.8	58.6	63.5	551.8
OTSU	88.9	78.1	80.8	3.0
Our Method	94.7	84.1	86.7	3.1

**Table 3:** The accuracy comparison of the cross-sectional area of the segmented hollowing from images.

	Relative Error in Area/%
Mean Method	70.3
Bimodal Thresholding Method	81.4
Maximum Entropy Method	80.2
OTSU	17.1
Our Method	9.7

## Data Availability

No new data were created or analyzed in this study. Data sharing is not applicable to this article.

## Abbreviations

The following abbreviations are used in this manuscript:

UAV	Unmanned Aerial Vehicle
PINNs	Physics-Informed Neural Networks
IR	Infrared
NDT	Non-Destructive Testing
IRT	Infrared Thermography
LSM	Level Set Method
PIRT	Passive Infrared Thermography
PBT	Progressive Background-aware Transformer
mAP	Mean Average Precision
MS-PINNs	Multi-Subnet Physics-Informed Neural Network
AD	Automatic Differentiation
PA	Pixel Accuracy
IoU	Intersection over Union
mIoU	Mean Intersection over Union
